# Disentangling the Complexity of a Hexa-Herbal Chinese Medicine Used for Inflammatory Skin Conditions—Predicting the Active Components by Combining LC-MS-Based Metabolite Profiles and *in vitro* Pharmacology

**DOI:** 10.3389/fphar.2018.01091

**Published:** 2018-10-05

**Authors:** Jennifer B. Chang, Majella E. Lane, Min Yang, Michael Heinrich

**Affiliations:** ^1^Research Group Pharmacognosy and Phytotherapy, UCL School of Pharmacy, London, United Kingdom; ^2^Department of Pharmaceutics, UCL School of Pharmacy, London, United Kingdom; ^3^Department of Pharmaceutical & Biological Chemistry, UCL School of Pharmacy, London, United Kingdom

**Keywords:** CCL17, Chinese herbal medicine formula, chemometric, HaCaT, inflammation, LC-MS-based metabolite profiles, partial least-squares regression

## Abstract

**Objectives:** The purpose of this study is to investigate the anti-inflammatory activity of a hexa-herbal Chinese formula (HHCF) using spontaneously immortalized human epidermal keratinocytes (HaCaT) and to predict the active components by correlating the LC-MS-based metabolite profiles of the HHCF and its 12 varied formulae with their anti-inflammatory activity using partial least-squares regression analysis.

**Methods:** The HHCF comprises the rootstock of *Scutellaria baicalensis, Rheum tanguticum, Sophora flavescens*, the root bark of *Dictamnus dasycarpus*, the bark of *Phellodendron chinense*, and the fruit of *Kochia scoparia* in equal proportions. Its 12 varied formulae were developed by uniform design with varied proportions of the component botanical drugs. The decoctions of the HHCF and its 12 varied formulae were profiled using liquid chromatography (LC) combined with triple quadrupole mass spectrometry (MS) and their effects on tumor necrosis factor (TNF)-α -plus-interferon (IFN)-γ-induced C-C motif chemokine ligand 17 (CCL17) production in HaCaT were investigated. Partial least-squares regression analysis was conducted to assess the relationship between the LC-MS-based metabolite profiles of the decoctions to anti-CCL17 production in HaCaT.

**Results:** Compounds with potential to promote anti-CCL17 production in HaCaT were identified (e.g., berberine, pyrogallol and catechin dimers) as a result of the developed model and their potential to act as anti-inflammatory agents were also supported by relevant literature.

**Conclusion:** This promising approach should assist in the screening process of active components from complex Chinese herbal preparations and will better inform the necessary pharmacological experiments to take forward.

## Introduction

Chinese medicine views a disease condition as the result of different syndromes and treats the diagnosed disorders using a combination of botanical drugs—a formula that has been optimized based on centuries of clinical experiences. The formulae of Chinese herbal medicine (CHM) act as mixture-based libraries for development of multicomponent therapeutic agents that may interact favorably with multiple targets, to achieve therapeutic effects with fewer side effects (Medina-Franco et al., 2013; Koeberle and Werz, 2014). Instead of isolating and testing pharmacological activities of individual chemical components of a CHM drugs or formulae, here we use a strategy in which we first want to understand the exact composition used in one specific preparation (in this case an aqueous extract). This strategy has been used far less commonly and offers the opportunity to understand the composition and the effects of the preparations used. Advancements in analytical techniques open up the possibility of profiling a multitude of small molecule metabolites in the complex CHM extracts. These fingerprints of CHM extracts can potentially be used to assess the composition of preparations and consistency of chemical constituents from batch-to-batch extracts and to ensure reproducible clinical effects by monitoring the bioactive components. Specifically, correlating metabolites profiles of CHM formulae to their bioactive effects using chemometrics has become an alternative approach to investigate the bioactive ingredients of CHM (Xu et al., 2014). For example, Wang et al. explored the bioactive components of a CHM formula by analyzing the relationship between the peaks area of prominent peaks in its HPLC fingerprints and the biological effects *in vivo* (Wang et al., 2014). While Su et al. explored the bioactive components of a CHM formula by analysing the relationship between the peak areas of prominent peaks in its GC fingerprints and the biological effects *in vitro* (Su et al., 2008).

In the present work, a method of predicting the active components in a Chinese herbal formula was used by correlating the metabolites in the LC-MS-based metabolite profiles of the Chinese herbal formula and related formulae to their respective levels of *in vitro* activity using chemometrics. In previous published works, only peak areas of characteristic peaks were used as the independent variables in building the multivariate regression models. Here, metabolites are selected based on their ion intensity levels in the extracts in descending order. Ion intensities of compounds in the LC-MS fingerprints were used as the independent variables that are more specific than peak areas in representing the metabolites in the extracts. The specific preparation is a hexa-herbal Chinese formula (HHCF) comprising rootstock of *Scutellaria baicalensis* Georgi (Lamiaceae; SCU), *Rheum tanguticum* Maxim. ex Balf. (Polygonaceae; RHE), *Sophora flavescens* Aiton (Fabaceae; SOP), root bark of *Dictamnus dasycarpus* Turcz. (Rutaceae; DIC), bark of *Phellodendron chinense* C. K. Schneid. (Rutaceae; PHE), and fruit of *Kochia scoparia* (L.) Schrad. (Amaranthaceae; KOC). The HHCF consists of four botanical drugs that are used in the “San Huang Xi Ji” formula. “San Huang Xi Ji” is a skin wash prepared by decocting equal amounts of PHE, RHE, SCU and SOP in water and is indicated for inflammatory skin conditions associated with pathogenic-heat, dampness and wind such as atopic dermatitis (Liang, 1993). In the HHCF, DIC and KOC are added to the “San Huang Xi Ji” formula in order to enhance the therapeutic effect. The actions of each botanical drugs in the HHCF according to the concepts of TCM are summarized in Table 1.

**Table 1:** Actions of botanical drugs in the HHCF.

**Pattern**	**Botanical drugs in the HHCF**
Clearing heat and dry-dampness	Dried rootstock of *Sophora flavescens* Aiton, Dried rootstock of *Scutellaria baicalensis* Georgi Dried bark of *Phellodendron chinense* C. K. Schneid.
Removing wind to stop itchiness	Dried root bark of *Dictamnus dasycarpus* Turcz. Dried fruit of *Kochia scoparia* (L.) Schrad.
Clearing heat and toxin	Dried rootstock of *Rheum tanguticum* Maxim. Ex Balf. Dried rootstock of *Scutellaria baicalensis* Georgi Dried bark *of Phellodendron chinense* C. K. Schneid. Dried root bark of *Dictamnus dasycarpus* Turcz.

To explore the active components of the HHCF, the major metabolites in the LC-MS-based metabolites profiles of the HHCF and its 12 varied formulae decoctions were correlated with their effects on TNF-α -plus-IFN-γ-induced CCL17 production in HaCaT, using partial least-squares regression (PLS-R).

## Materials and methods

### Materials

All botanical drugs were purchased from commercial Chinese herbal medicine stores in China. SCU, RHE, SOP, DIC, KOC, and PHE were sourced from Hebei (Chengde), Gansu (Maqu county), Hebei (Chengde), Liaoning (Anshan), Hebei (Chengde), and Sichuan (Dujiangyan), respectively, and were authenticated by the first author based on her experience with CHMs. Samples were deposited at the School of Pharmacy Medicinal Plant Herbarium and are numbered as JC1–6. MS grade formic acid and LC-MS grade acetonitrile were obtained from Sigma-Aldrich and LC-MS grade water was obtained from Fisher Scientific. HaCaT were obtained from Cell Lines Service, Eppelheim, Germany. Dulbecco's modified Eagle medium (DMEM), Ca^2+^ and Mg^2+^ -free phosphate buffer saline (PBS) and 100 U/mL penicillin and 100 μg/mL streptomycin were obtained from Gibco. 10% heat inactivated fetal bovine serum, DMSO and 3-(4,5-dimethylthizaol-2-yl)-2,5-diphenyltetrazolium bromide (MTT) were obtained from Sigma-Aldrich. Human TNF-α and IFN-γ were obtained from Peprotech. The human CCL17 DuoSet enzyme linked-immunosorbent assay (ELISA) kit was obtained from R&D Systems.

### Preparation of the HHCF and the 12 varied formulae decoctions

All botanical drugs, except SCU, were blended into a powder and SCU was cut into small blocks of 1 × 1 cm (powdered SCU will result in a too viscous extract that cannot be filtered) before the decoction process. For the HHCF decoction, the same ratio of each botanical drug (i.e., SCU, RHE, SOP, DIC, PHE, and KOC) was used. A six-factor, 12-level uniform design was applied to establish differences among the 12 varied formulae of the HHCF (i.e., V1-V12; Table 2). Table 2 was developed based on the U_12_ (12^5^) uniform deign table and method described in Fang (1994).

**Table 2:** Percentage of 6 botanical drugs in the 12 varied formulae of the HHCF under uniform design.

	**SOP (%)**	**SCU (%)**	**PHE (%)**	**RHE (%)**	**DIC (%)**	**KOC (%)**
V1	47	17	12	4	1	19
V2	34	12	8	18	4	25
V3	27	6	1	53	3	11
V4	22	1	31	5	12	29
V5	18	33	9	13	10	17
V6	14	19	3	41	10	12
V7	12	10	39	3	20	17
V8	9	3	20	18	31	19
V9	7	51	3	21	13	5
V10	5	25	46	1	19	5
V11	3	14	23	13	42	6
V12	1	6	10	38	43	2

For each formula, botanical drugs were first macerated in distilled water (at a volume of 5 fold the dry weight of botanical drug used) for 1 h and then heated under reflux for 95 min. The extracted solution was filtered through nylon cloth of pore size ~0.1 mm, followed by centrifugation at 10,000 rpm for 5 min. The collected supernatant was lyophilized.

### LC-MS/MS profiling of the HHCF and its 12 varied formulae

Lyophilized decoctions of the HHCF and its 12 varied formulae were dissolved in LC-MS grade water to achieve a concentration of 20 mg/mL, centrifuged at 10,000 rpm for 10 min and filtered through 0.22 μm filter membrane before analysis. They were subsequently profiled by LC-MS using the method described in the “LC-MS/MS analysis” section of the previous publication (Chang et al., 2016).

### Partial least-squares regression (PLS-R) analysis

The abundance of major metabolites in the HHCF and its 12 varied formulae were used as the independent variables. The reciprocal levels of CCL17 produced by HaCaT after treatment with the HHCF and its 12 varied formulae at a concentration of 60 μg/mL were used as the dependent variables. At this concentration, all tested samples demonstrated statistically significant CCL17 inhibition in HaCaT stimulated with TNF-α-plus-IFN-γ. The independent and dependent variables were mean-centered and scaled and were subsequently imported to JMP Pro 12 software from SAS to build the PLS-R model. Leave-one-out cross validation was carried out to select the optimal number of latent variables for the PLS-R analysis based on the root-mean-square error of cross-validation (RMSECV) value.

### Cell culture

The HaCaT (Boukamp et al., 1988) were cultured in DMEM containing 10% heat inactivated fetal bovine serum and 100 U/ml penicillin and 100 μg/mL streptomycin, in 5% CO_2_ at 37 (Turksen, 2004).

### Enzyme linked immunosorbent assay (ELISA)

HaCaT were seeded into a 96-well plate (200 μl per well of 2 × 10^4^ cells/mL). After 24 h, the medium was replaced with serum-free medium and cells were cultured for another 24 h. The medium was then removed and cells were treated with fresh serum-free medium containing the test sample. After 5 min, 30 ng/mL TNF-α and 30 ng/mL IFN-γ were added and the cells were cultured for 24 h. After incubation, the medium was collected and analyzed for CCL17 by ELISA according to the manufacturer's instruction (Fujita et al., 2011).

### Cell viability assay

Cells were assessed for viability using the 3-(4,5-dimethylthizaol-2-yl)-2,5-diphenyltetrazolium bromide (MTT) assay. HaCaT were seeded into a 96-well plate (200 μL per well of 2 × 10^4^ cells/mL). After 24 h, the medium was replaced with serum-free medium and cells were cultured for another 24 h. The medium was then replaced with fresh medium containing the test sample. After incubation for 24 h, the medium was removed and cells were washed once with PBS and exposed to 0.5 mg/mL of MTT for 3 h, in 5% CO_2_ at 37°C. Cells were then washed once with PBS and the formazan precipitate was dissolved in DMSO (200 μL) and the absorbance at 570 nm was measured using a microplate reader. The percentage of cell viability was assessed as [mean absorbance in tested wells]/[mean absorbance in control wells] × 100 (Qi et al., 2009). Assays were performed with two replicates in three independent experiments (*n* = 3).

## Results and discussion

### HHCF and its 12 varied formulae

**Figure 1:**
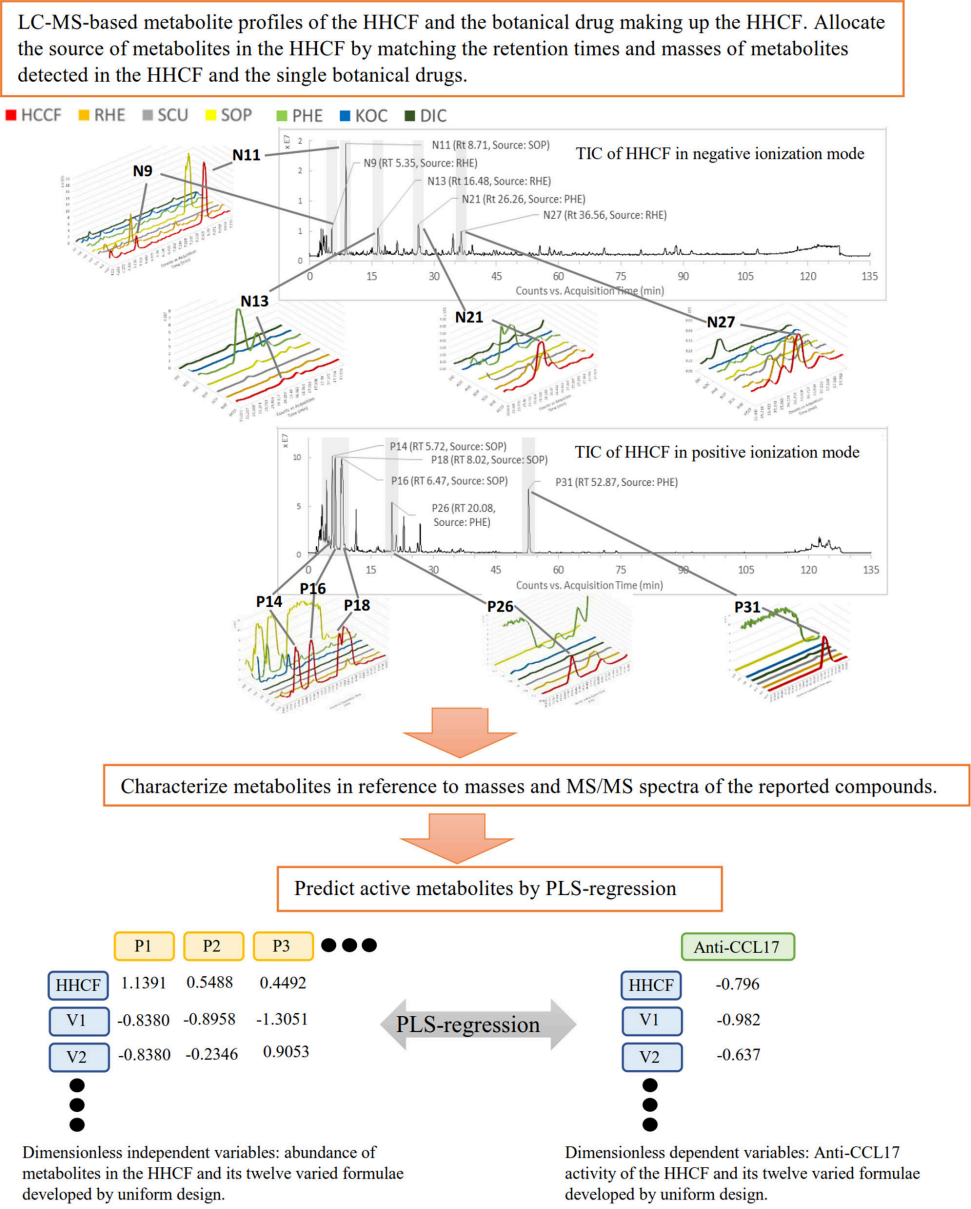
The schematic diagram of the proposed approach. N9, Nll, N13, N21 and N27, and P14, P16, PIS, P26, and P31 are the top 5 most abundant metabolites in the HHCF in negative and positive ionization mode, respectively. Chemical structures of these metabolites are shown in Figure 2.

The findings of this study underline the power of LC-MS-based metabolite profiling, coupled with PLS-R to predict potential active components in CHM decoctions. The schematic diagram of this study is shown in Figure 1. The PLS-R model was developed based on the hypothesis that *in vitro* activity of CHM decoctions varied with differences in chemical components. To create the differences, 12 varied formulae of the HHCF were developed by a uniform mixture design approach. Uniform mixture design seeks to spread the experimental points uniformly over the design space and hence, facilitate the exploration of the relationship between the *in vitro* biological response and the chemical components with fewer number of runs when compared to other experimental design methods such as factorial design (Liang et al., 2001; Fang and Lin, 2003).

### LC-MS/MS metabolite profiling

**Figure 2:**
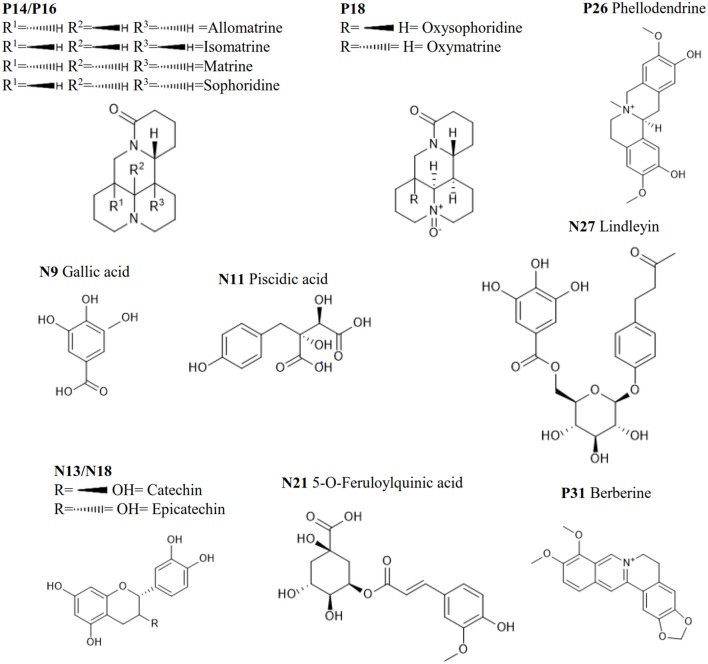
The top 5 most abundant metabolites in the HHCF in positive and negative ionization mode.

In previous publication (Chang et al., 2016), the chemical compounds characterized in the HHCF in both positive (Table 3A) and negative (Table 3B) modes were putatively identified based on mass measurement and characteristic fragment ions and by reference to the mass and MS/MS spectra of reported compounds. The sources of these compounds were defined by matching the retention times and masses of ions detected in the HHCF decoction and the single botanical drug decoctions, using an in-house developed EXCEL template. Figures S1, S2 show the total ion count (TIC) chromatograms of the single botanical drug decoctions (i.e., DIC, KOC, PHE, RHE, SCU, and SOP) in positive and negative modes, respectively. The top 5 most abundant compounds in the HHCF in positive ionization mode are P14(allomatrine/ isomatrine/matrine/sophoridine), P16 (allomatrine/isomatrine/matrine/sophoridine), P26 (phellodendrine), and P31 (berberine). The top 5 most abundant compounds in the HHCF in negative ionization mode are N9 (gallic acid), N11(piscidic acid), N13 (catechin/epicatechin), N18 (catechin/epicatechin), N21(5-*O*-feruloylquinic acid), and N27 (lindleyin). The chemical structures of these compounds are shown in Figure 2.

**Table 3A:** Putatively identified compounds in the HHCF by LC-MS/MS in positive ionization mode.

**Rt (min)**	**No**.	***m/z***	**Adduct ion(s)**	**Source**	**Identity (CAS number)**
3.36	P1	191	[M+H]^+^	SOP	Cytisine (485-35-8)
	P2	196	[M]^+^	PHE	Atraric acid (4707-47-5)
	P3	205	[M+H]^+^	SOP	N-Methylcytisine (6220-47-9)
	P4	215	[M]^+^	RHE	Mecoprop (93-65-2)
	P5	261	[M+H]^+^	SOP	Baptifoline (732-50-3)
3.75	P6	265	[M+H]^+^	SOP	5α-Hydroxymatrine (3411-37-8)/9α-Hydroxymatrine (88509-92-6)
4.15	P7	265	[M+H]^+^	SOP	14β-Hydroxymatrine (183074-18-2)
4.40	P8	180	[M]^+^	PHE	Candicine (6656-13-9)
4.75	P9	245	[M+H]^+^	SOP	Anagyrine (486-89-5)
	P10	265	[M+H]^+^	SOP	5α-Hydroxymatrine (3411-37-8)/9α-Hydroxymatrine (88509-92-6)
5.00	P11	247	[M+H]^+^	SOP	Isosophocarpine (68398-59-4)
5.52	P12	263	[M+H]^+^	SOP	(-)-9α-hydroxy-7, 11-dehydromatrine (1257392-34-9)
5.72	P13	192	[M+H]^+^	PHE	Noroxyhydrastinine (21796-14-5)
	P14	249	[M+H]^+^	SOP	Allomatrine (641-39-4)/Isomatrine (17801-36-4)/Matrine (519-02-8)/Sophoridine (6882-68-4)
6.47	P15	247	[M+H]^+^	SOP	Sophocarpine (6483-15-4)
	P16	249	[M+H]^+^	SOP	Allomatrine (641-39-4)/Isomatrine (17801-36-4)/Matrine (519-02-8)/Sophoridine (6882-68-4)
8.02	P17	263	[M+H]^+^	SOP	Oxysophocarpine (26904-64-3)
	P18	265	[M+H]^+^	SOP	Oxymatrine (16837-52-8)/Oxysophoridine (1217501-78-4)
	P19	266	[M+NH_4_]^+^	SOP	Lupanine (550-90-3)
10.42	P20	247	[M+H]^+^	SOP	(+)-7,11-Dehydromatrine (46862-63-9)
11.30	P21	263	[M+H]^+^	SOP	9α-Hydroxysophocarpine (220961-52-4)
11.48	P22	263	[M+H]^+^	SOP	Leontalbinine N-oxide (147731-96-2)
	P23	265	[M+H]^+^	SOP	Oxymatrine (16837-52-8)/Oxysophoridine (1217501-78-4)
11.88	P24	243	[M+H]^+^ /	DIC	Dasycarpusenester A (1419709-60-6)
			[M]^+^		O-Ethylnor-γ-fagarine (105988-99-6)
16.77	P25	314	[M]^+^	PHE	(-)-Oblongine (152230-57-4)
20.08	P26	342	[M+H]^+^	PHE	Phellodendrine (6873-13-8)
22.08	P27	344	[M+H]^+^	PHE	Tembetarine (18446-73-6)
22.91	P28	342	[M+H]^+^	PHE	Magnoflorine (2141-09-5)
26.84	P29	314	[M+H]^+^	PHE	Evoeuropine (524-20-9)
31.32	P30	356	[M+H]^+^	PHE	Menisperine (25342-82-9)
52.87	P31	336	[M]^+^	PHE	Berberine (2086-83-1)

**Table 3B:** Putatively identified compounds in the HHCF by LC-MS/MS in negative ionization mode.

**Rt (min)**	**No**.	***m/z***	**Adduct ion(s)**	**Source**	**Identity (CAS number)**
2.34	N1	193	[M-H]^−^	SCU	Glucuronic acid (6556-12-3)
2.46	N2	191	[M-H]^−^	PHE	Quinic acid (77-95-2)
	N3	223	[M-H]^−^	SOP	Sinapic acid (530-59-6)
3.50	N4	191	[M-H]^−^	PHE	Citric acid (77-92-9)
	N5	331	[M-H]^−^	RHE	Galloylglucose [i.e., 1-*O*-Galloyl-β-D-glucose (13405-60-2) or
4.08	N6	331	[M-H]^−^	RHE	6-*O*-Galloyl-β-D-glucose (34781-46-9)]/Glucopyranosyloxyl gallic acid [i.e. Gallic
4.61	N7	331	[M-H]^−^	RHE	acid-3-O-β-D-glucoside (91984-84-8) or Gallic acid-4-O-β-D-glucoside (84274-52-2)]
5.35	N8	125	[M-H]^−^	RHE	Pyrogallol (87-66-1)
	N9	169	[M-H]^−^	RHE	Gallic acid (149-91-7)
	N10	331	[M-H]^−^	RHE	Galloylglucose [i.e. 1-*O*-Galloyl-β-D-glucose (13405-60-2) or 6-*O*-Galloyl-β-D-glucose (34781-46-9)]/Glucopyranosyloxyl gallic acid [i.e. Gallic acid-3-O-β-D-glucoside (91984-84-8) or Gallic acid-4-O-β-D-glucoside (84274-52-2)]
8.71	N11	255	[M-H]^−^	SOP	Piscidic acid (35388-57-9)
13.88	N12	577	[M-H]^−^	RHE	Procyanidin B (15514-06-4)
16.48	N13	289	[M-H]^−^	RHE	Catechin (154-23-4)
	N14	353	[M-H]^−^	PHE	Chlorogenic acid (327-97-9)
18.15	N15	367	[M-H]^−^	PHE	3-*O*-Feruloylquinic acid (1899-29-2)
21.10	N16	325	[M-H]^−^	RHE	4-(4′-Hydroxylphenyl)-2-butanone 4′-O-β-D-glucoside (38963-94-9)
	N17	415	[M+Na-2H]^−^	RHE	6-Hydroxymusizin-8-O- β-D-glucoside(23566-96-3)
22.77	N18	289	[M-H]^−^	RHE	Epicatechin (490-46-0)
	N19	337	[M-H]^−^	PHE	*p*-coumaroylquinic acid (87099-71-6/93451-44-6)
24.72	N20	303	[M-H]^−^	SCU	2′,3,5,6′,7-Pentahydroxyflavanone (1402054-86-7/80366-15-0)
26.26	N21	367	[M-H]^−^	PHE	5-*O*-Feruloylquinic acid (40242-06-6)
26.65	N22	389	[M-H]^−^	RHE	Resveratrol-4′-O-β-D-glucoside (38963-95-0)/Resveratrol 3-O-β-glucoside (27208-80-6)
32.06	N23	301	[M-H]^−^	SCU	3,5,7,2′,6′-Pentahydroxyflavone (92519-95-4)
34.57	N24	441	[M-H]^−^	RHE	Epicatechin 3-O-gallate (1257-08-5)
	N25	477	[M-H]^−^	RHE	Isolindleyin (87075-18-1)
	N26	547	[M-H]^−^	SCU	Chrysin-6-C-arabinosyl-8-C-glucoside (185145-33-9/ 1884390-97-9)
36.56	N27	477	[M-H]^−^	RHE	Lindleyin (59282-56-3)
	N28	545	[M-H]^−^	RHE	Rhein-8-O-D-[6′-O-(3″-methoxylmalonyl)] glucoside (1333328-11-2)
37.25	N29	547	[2M-H]^−^	SCU	Chrysin-6-C-glucosyl-8-C-arabonoside (185145-34-0/ 1884390-98-0)
38.43	N30	541	[M-H]^−^	RHE	Resveratrol-4′-O-β-D-(2″-O-galloyl) glucoside (105304-51-6)
39.20	N31	541	[M-H]^−^	RHE	Resveratrol-4′-O-β-D-(6″-O-galloyl) glucoside (64898-03-9)
45.08	N32	301	[M-H]^−^	SCU	Trihydroxy-methoxyflavanone (92519-96-5)
46.91	N33	431	[M-H]^−^	RHE	Emodin-1-O-β-D-glucoside (38840-23-2)/Emodin-8-O-β-D-glucoside (23313-21-5)/Aloe-emodin 8-O-β-D-glucoside (33037-46-6)/Aloe-emodin-3-CH_2_-O-β-D-glucoside (50488-89-6)
55.48	N34	481	[M+Cl]^−^	SOP	(-)-Maackiain-3-O-glucoside (6807-83-6)
58.94	N35	431	[M-H]^−^	RHE	Emodin-1-O-β-D-glucoside (38840-23-2)/Emodin-8-O-β-D-glucoside (23313-21-5)/Aloe-emodin 8-O-β-D-glucoside (33037-46-6)/Aloe-emodin-3-CH_2_-O-β-D-glucoside (50488-89-6)
63.95	N36	233	[M-H]^−^	RHE	(5Z)-6-Hydroxy-3,4-dioxo-6-phenyl-5-hexenoic acid (NA)
71.02	N37	269	[M-H]^−^	SCU	5,6,7-Trihydroxyflavone (491-67-8) OR 5,7,8-Trihydroxyflavone (4443-09-8)

**Figure 3:**
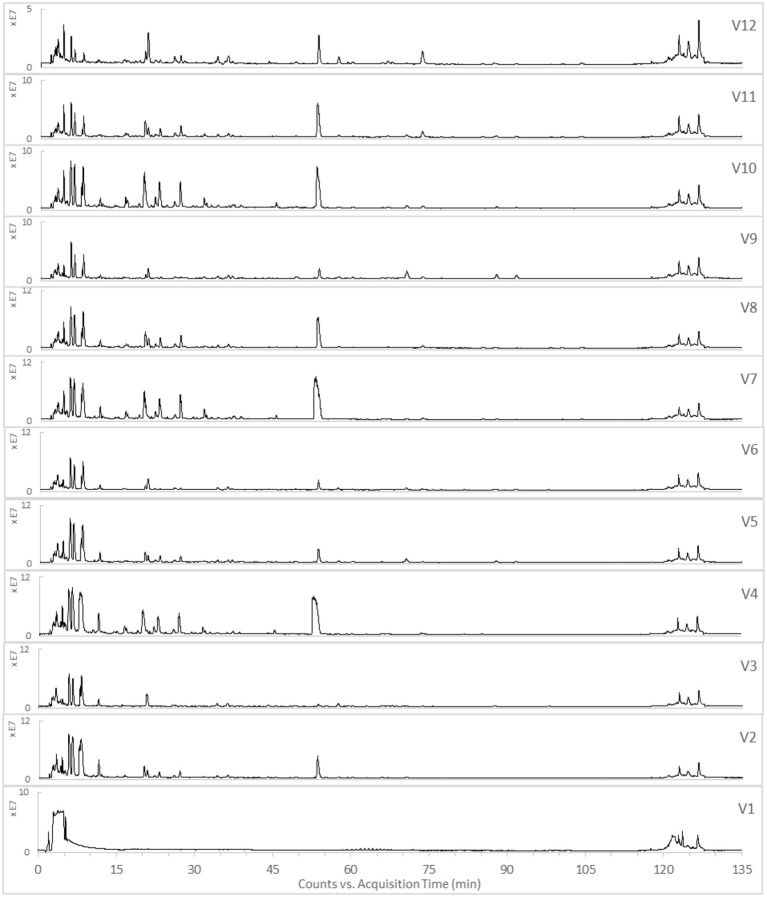
TICs of the 12 varied formulae (Vl-V12) of the HHCF in positive ionization mode.

**Figure 4:**
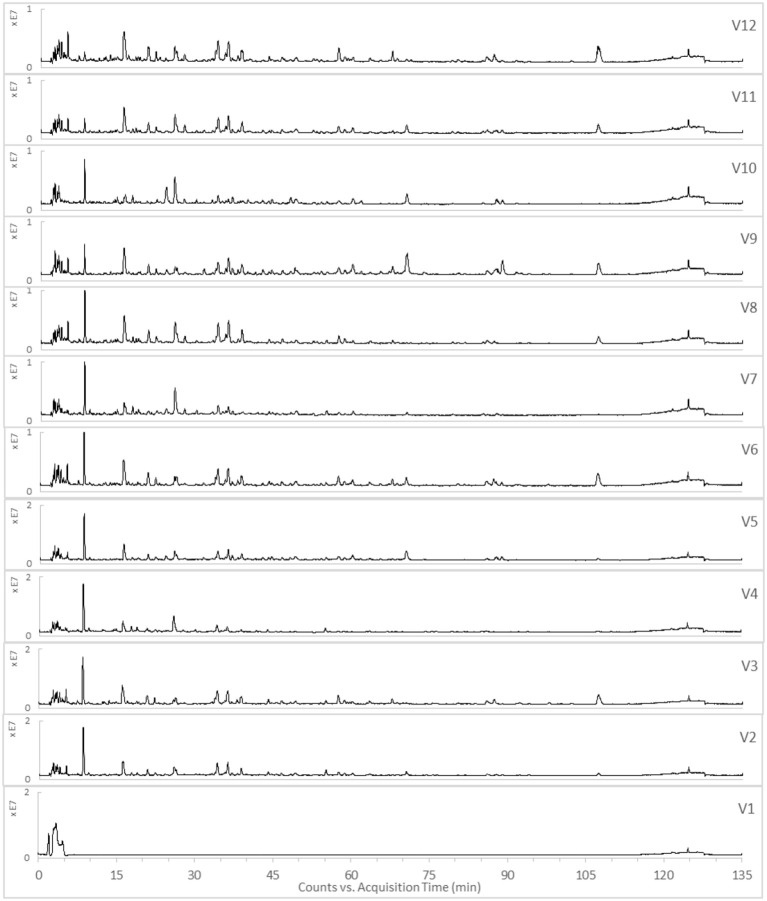
TICs of the 12 varied formulae (Vl-V12) of the HHCF in negative ionization mode.

The TICs of the 12 varied formulae of the HHCF in positive and negative ionization modes are shown in Figures 3,4, respectively. The abundance of the characterized metabolites in the HHCF and its 12 varied formulae decoctions are shown in Table S1. These values of abundance were mean-centered and scaled (Table S2) and were used as the independent variables for building the PLS-R model.

### Effects of the HHCF and its 12 varied formulae on CCL17 production in HaCaT stimulated with TNF-α-plus-IFN-γ

**Figure 5:**
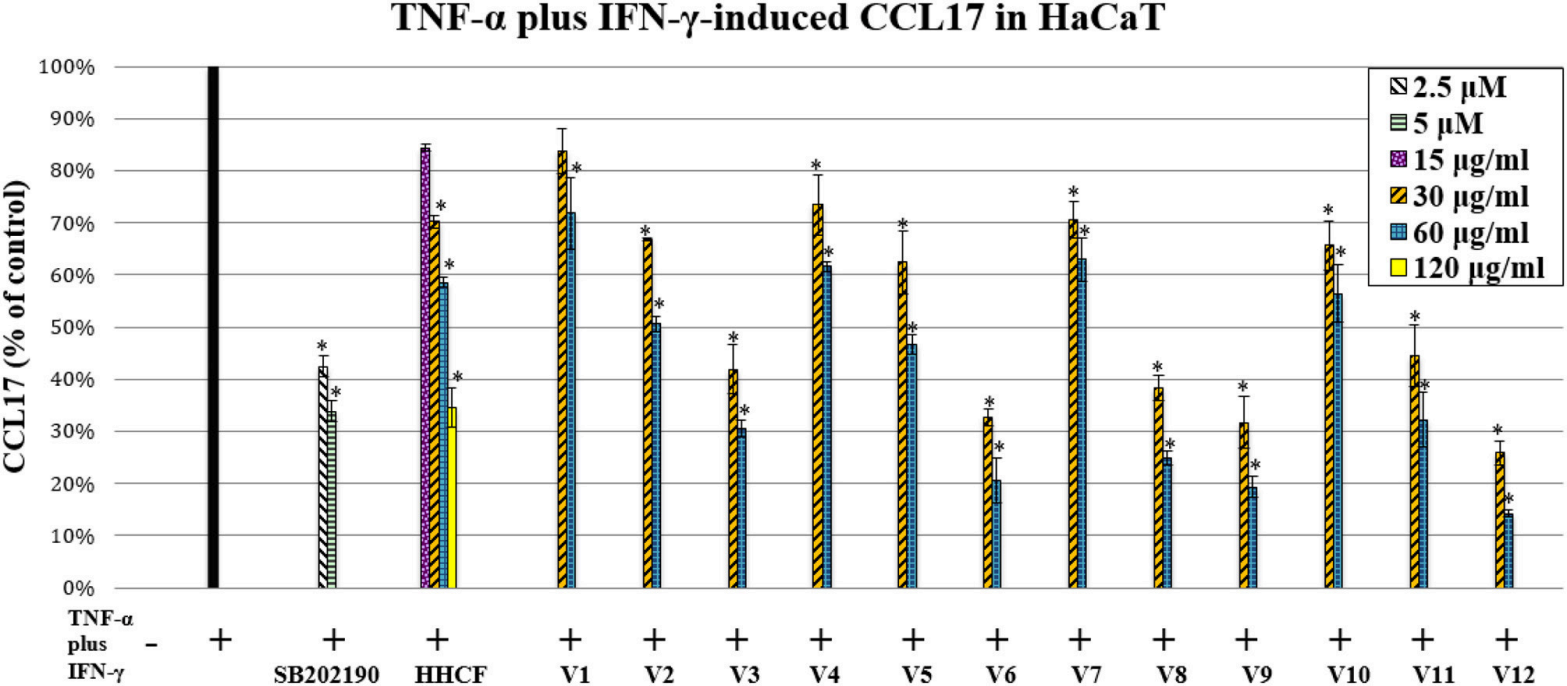
Effect of the HHCF (15, 30, 60, and 120 μ/ml) and its twelve varied formulae (Vl-V12; 30 and 60 μ/ml), SB202190 monohydrochloride hydrate (positive control; 2.5 and 5 μ) on TNF-a plus IFN-y-induced CCL17 production in HaCaT. Data are represented as mean ± standard error of three independent experiments (*n* = 3). Statistical significance was determined using one-way analysis of variance with Dunnett's multiple comparisons test. *p < 0.05 vs. TNF-a plus IFN-y treatment alone.

CCL17 have previously been demonstrated to be linked to the pathogenesis of atopic dermatitis. They have been detected in lesional AD skin but not in normal or non-lesional AD tissue (Vestergaard et al., 2000; D'Ambrosio et al., 2002). In addition, increased serum levels of CCL17 in individuals with AD were correlated with disease severity (Kakinuma et al., 2001). CCL17 are ligands for the CC chemokine receptor 4 (CCR4) that are primarily expressed on Th2 lymphocytes (Saeki and Tamaki, 2006). Hence, CCL17 contribute to the infiltration of Th2 lymphocytes in skin inflammation sites. The HCCF inhibited the production of CCL17 in HaCaT stimulated with TNF-α-plus-IFN-γ (Figure 5). To investigate which compounds in the LC-MS metabolites profile of the HHCF were most likely to be the contributors to the observed CCL17 inhibition, the effects of the 12 varied formulae (V1-V12) of the HHCF against CCL17 production in HaCaT were also tested. The HHCF decoction and its 12 varied formulae showed different degree of CCL17 inhibition in the HaCaT stimulated with TNF-α-plus-IFN-γ (Figure 5). Results from the MTT assay demonstrated that the decreased CCL17 levels were not due to any toxic effects (Figure S3) of the samples on the cells.

The levels of CCL17 produced by TNF-α-plus-IFN-γ-stimulated HaCaT after treatment with HHCF and its 12 varied formulae (V1-V12) decoctions at a concentration of 60 μg/ml were used as the dependent variables for building the PLS-R model. A lower absolute value of CCL17 represents a higher inhibition effect, thus, a reciprocal was applied to the obtained data. These data were then centered and scaled before model building (Table S3).

### Prediction of potential active compounds in HHCF using PLS-R

Compounds in the LC-MS metabolites profile of the HHCF that were most likely to be the contributors to the observed CCL17 inhibition were predicted using PLS-R. PLS-R analysis was used as (1) the number of dependent variables (response; Table S3) was less than the number of independent variables (also known as predictor variable; Table S2) and (2) this approach uses linear combinations of the independent variables and avoids the multicollinearity problem among the variables (Miller and Miller, 2010). The number of latent variables for the PLS-R analysis was selected based on the RMSECV value and the percentage of variance explained by the PLS-R model. The number of latent variables for the PLS-R analysis was selected to be 9, representing the point of final drop in the prediction error before the curve reaches a plateau (Figure S4). The 9 latent variables in the PLS-R model explained 100% of the variation and 88.19% of the variance was explained by the regressors (Table S4). Table 4 shows the regression coefficient of the independent variables calculated using PLS-R analysis. The positive and negative values of the regression coefficient (RC) indicate a positive and negative contribution to the response (i.e., CCL17 inhibition), respectively. Additionally, a higher absolute value represents a larger contributory effect (Wang et al., 2014). The top five contributors in the HHCF that promote CCL17 inhibition in the PLS-R model were P31 (berberine), N8 (pyrogallol), N12(catechin dimers), N16 (4-(4′-hydroxyphenyl)-2-butanone 4′-O-β-D-glucoside) and N31 (resveratrol 4′-O-β-D-(6″-O-galloyl) glucoside). Other glycosides of resveratrol i.e., N30 (resveratrol-4′-O-β-D-(2″-O-galloyl) glucoside) and N22 (resveratrol-4′-O-β-D-glucoside OR resveratrol 3-O-β-glucoside) in the HHCF are also important contributors to the CCL17 inhibition, ranking 7th and 8th, respectively. Of these, berberine, pyrogallol, catechin dimers, and resveratrol 3-O-β-glucoside have shown anti-inflammatory effects in various studies. Berberine was observed to inhibit the production of proinflammatory cytokines interleukin (IL)-6 and chemokines IL8 in HaCaT stimulated with sulfur mustard (Lang et al., 2018) and their anti-inflammatory effects have been linked to the inhibition of the nuclear factor-κB (NF-κB) signaling pathway (Li et al., 2016). Catechin dimers (Andre et al., 2012) and resveratrol 3-O-β-glucoside (Potapovich et al., 2011) have also been shown to inhibit NF-κB activation in TNF- α-stimulated-NF-κB/SEAP (Secreted alkaline phosphatase) HEK 293 cell lines and TNF-α-plus-IFN-γ-stimulated primary human keratinocytes. Pyrogallol was shown to inhibit mRNA expression of pro-inflammatory cytokines IL-6, chemokines (IL-8, CXCL1, and CXCL3) and Intercellular adhesion molecules-1 (ICAM-1) in cystic fibrosis bronchial epithelial cell lines (IB3-1 cells) stimulated with *P. aeruginosa* PAO1 (Nicolis et al., 2008). Thus, these suggest the underlying mechanisms for their respective potential anti-inflammatory roles in the HHCF decoction.

**Table 4:** Relevance [regression coefficient (RC)] between the chemical compounds putatively identified in the LC-MS profile of the HHCF decoction and CCL17 response.

**Ingredients**	**PLS-RC**	**Ingredients**	**PLS-RC**
P1	−0.0117	N1	−0.0655
P2	−0.0417	N2	0.0559
P3	0.0001	N3	0.0469
P4	0.0722	N4	−0.0379
P5	0.0071	N5	0.0724
P6	−0.1063	N6	0.0036
P7	−0.1002	N7	0.0763
P8	−0.0453	N8	0.0842
P9	−0.02	N9	0.0513
P10	−0.0445	N10	−0.026
P11	−0.0627	N11	−0.0415
P12	−0.0358	N12	0.0828
P13	0.0183	N13	0.0491
P14	0.0047	N14	−0.0203
P15	0.0205	N15	−0.0309
P16	0.0184	N16	0.0947
P17	0.0348	N17	−0.0623
P18	0.0066	N18	−0.0331
P19	0.005	N19	−0.0807
P20	−0.0046	N20	−0.0957
P21	−0.0263	N21	−0.0066
P22	−0.0186	N22	0.0726
P23	−0.0097	N23	0.0131
P24	0.0276	N24	−0.0403
P25	−0.0003	N25	−0.0759
P26	−0.0119	N26	−0.0036
P27	0.0069	N27	0.0685
P28	−0.0128	N28	−0.0059
P29	0.0016	N29	0.0273
P30	0.0149	N30	0.0742
P31	0.0823	N31	0.0765
		N32	−0.0016
		N33	−0.0114
		N34	−0.0237
		N35	0.0212
		N36	−0.0144
		N37	−0.0658

*The top five contributors in the HHCF toward the CCL17 inhibition in the PLS-R model were underlined*.

## Conclusion

In conclusion, an approach to predict potential active components in a CHM formula was demonstrated by correlating the LC-MS-based metabolite profiles of CHM formulae to their anti-inflammatory activities based on chemometrics. The results suggested that berberine, pyrogallol, catechin dimers, 4-(4′-hydroxyphenyl)-2-butanone 4′-O-β-D-glucoside and resveratrol 4′-O-β-D-(6″-O-galloyl) glucoside are the core anti-CCL17 bioactive ingredients in the HHCF. Further evaluation and validation of the activities of the predicted active components may support the application of metabolite profiling of a CHM formula as a quality control tool. This approach might also assist in the optimization of CHM formulae and drug discovery. Though the *in vitro* experimental studies were purely exploratory, they also indicate potential areas for further research of the HHCF as a botanical remedy for treatment of skin inflammation.

The strategy employed in this research can facilitate a better understanding of complex multiherbal preparations commonly used not only in TCM but also in other local and traditional medicines. While still time consuming it offers a strategy to clearly define the chemical basis of a complex preparation with regards to the preparation's pharmacological (or toxicological) activity.

## Author contributions

This study is a part of JC's Ph.D. thesis defended in 2017. MH, JC, and ML designed the strategy of research, MH and ML supervised the project as first and second supervisor, respectively. JC conducted the experiments and analyzed the data. MY supervised the MS-based experiments. All authors read and commented on earlier drafts of the MS.

### Conflict of interest statement

JC was a self-funded Ph.D. student in MH's and ML's group. The authors declare that the research was conducted in the absence of any commercial or financial relationships that could be construed as a potential conflict of interest. The reviewer WZ and handling Editor declared their shared affiliation.

## Abbreviations

CHM: Chinese herbal medicine
DIC: the root bark of *Dictamnus dasycarpus* Turcz.
HaCaT: spontaneously immortalized human epidermal keratinocytes
HHCF: hexa-herbal Chinese formula
KOC: the fruit of *Kochia scoparia* (L.) Schrad.
PHE: the bark of *Phellodendron chinensis* C.K. Schneid.
PLS-R: partial least-squares regression
RC: regression coefficient
RHE: the rootstock of *Rheum tanguticum* Maxim. ex Balf.
SCU: the rootstock of *Scutellaria baicalensis* Georgi
SOP: the rootstock of *Sophora flavescens* Aiton.
