# Supplementary material for: Disentangling the Complexity of a Hexa-Herbal Chinese Medicine Used for Inflammatory Skin Conditions—Predicting the Active Components by Combining LC-MS-Based Metabolite Profiles and in vitro Pharmacology
Source: Front Pharmacol. 2018 Oct 5;9:1091. doi: 10.3389/fphar.2018.01091 (PMC6182074; doi:10.3389/fphar.2018.01091)
Supplement: Supplementary file 1 [file Data_Sheet_1.PDF]

Supplementary Material

Disentangling the complexity of a hexa-herbal Chinese Medicine used for inflammatory skin conditions – predicting the active components by combining LC-MS-based metabolite profiles and in vitro pharmacology

Jennifer B. Chang*, Majella E. Lane, Min Yang, Michael Heinrich

*** Correspondence:** Prof. Michael Heinrich: m.heinrich@ucl.ac.uk

Table S1. Abundance of 68 putatively identified compounds in the LC-MS profile of the HHCF and its twelve varied formulae. (V1-V12 represented the twelve varied formulae. P1-P31 and N1-N37 represent compounds putatively identified in the HHCF LC-MS profile obtained under positive and negative ionization modes, respectively. The putatively assigned identity of P1-P31 and N1-N37 compounds can be found in Table 2a and 2b, respectively.)

	P1	P2	P3	P4	P5	P6	P7	P8	P9	P10	P11	P12	P13	P14	P15	P16
**HHC**	89035	216562	44243	53048	10126	12664	641651	310745	81570	462085	12740	394883	83105	445722	137959	199138
**V1**	0	0	23199	0	0	70250	702504	118044	26131	702504	66351	0	0	41775	16436	41775
**V2**	0	99125	55143	41681	17327	23399	762820	186073	13211	722600	14370	799841	0	0	198155	358391
**V3**	64590	63805	39878	11580	65846	83922	59147	346962	35224	56576	19256	168799	0	0	111504	225658
**V4**	80216	6063	87306	43130	91286	57265	895249	0	21062	205382	39368	505680	0	0	225202	347523
**V5**	91490	157505	40060	44609	15783	18314	571302	253922	76131	427386	10871	386882	62550	0	242669	326652
**V6**	0	0	26716	0	11799	89139	358802	855832	31759	220610	24133	139511	0	0	134470	196092
**V7**	116464	251495	57328	0	23750	13398	548453	372570	10462	334902	17860	0	0	0	258060	297261
**V8**	0	0	19960	0	90893	58577	439297	273033	56616	283072	12665	0	163539	508990	188678	243533
**V9**	48790	46534	18945	73119	93908	59457	196029	427329	19656	97984	12181	0	0	0	120683	136797
**V10**	0	503470	25833	42402	11966	83362	398161	375340	67424	216748	89297	0	0	408593	215301	269645
**V11**	0	298310	13619	54157	68699	36190	177370	288892	23274	99047	76538	0	0	0	104361	113296
**V12**	0	102963	42599	81177	0	11739	68806	128674	0	7419	10857	0	48925	675806	141068	158252

	P17	P18	P19	P20	P21	P22	P23	P24	P25	P26	P27	P28	P29	P30	P31	N1
**HHC**	196110	317140	51240	14745	23326	97353	109318	85942	20733	161799	11643	660146	750636	67973	12607	14626
**V1**	0	16742	2982	16436	0	0	16742	0	0	0	0	0	0	0	0	0
**V2**	529478	0	90395	15229	37707	17580	206586	0	14611	144266	58172	0	0	37032	16700	11438
**V3**	110010	291226	41483	73096	28065	27380	157744	0	24778	74807	26183	35953	26138	27943	3647	0
**V4**	163257	470793	75174	24751	22505	34573	146637	3558	0	509407	7716	31187	178367	0	15614	0
**V5**	141722	365296	56406	10884	17801	10121	112755	0	90819	148444	83791	0	591471	88034	13959	0
**V6**	202795	40681	4545	23873	13869	13869	139049	0	4367	291243	13717	65968	123929	5760	21907	0
**V7**	353606	0	75806	17203	17408	12198	133647	98283	44680	254579	47412	453414	360162	115929	0	0
**V8**	180737	444947	64667	12331	9963	55127	756259	0	17671	228695	14284	124442	170075	205506	20302	0
**V9**	108629	24802	0	36110	0	19267	341392	62139	13550	352302	12949	87565	98250	4510	16570	0
**V10**	172644	462382	67806	82792	9484	70254	928720	111221	93499	286819	82030	0	340257	918678	0	0
**V11**	653153	15316	2012	13952	4212	4212	38977	0	15252	200571	15079	979958	119846	114376	16370	0
**V12**	133011	4832	0	4429	0	0	20410	7431	0	360066	25646	131451	227903	25808	23148	0

Table S1. Abundance of 68 putatively identified compounds in the LC-MS profile of the HHCF and its twelve varied formulae. (V1-V12 represented the twelve varied formulae. P1-P31 and N1-N37 represent compounds putatively identified in the HHCF LC-MS profile obtained under positive and negative ionization modes, respectively. The putatively assigned identity of P1-P31 and N1-N37 compounds can be found in Table 2a and 2b, respectively.)

	N2	N3	N4	N5	N6	N7	N8	N9	N10	N11	N12	N13	N14	N15	N16	N17
**HHC**	35138	7964	37172	28289	64187	39018	14147	52216	23918	843196	14754	189793	20057	32138	12317	11088
**V1**	0	0	1530	1501	0	0	0	0	0	0	0	0	0	0	0	0
**V2**	25628	0	34711	0	69694	34036	0	29283	23633	0	0	192383	8507	12010	10062	1508
**V3**	85915	30875	36865	16676	14509	0	0	11445	5255	6724	0	8006	3676	7557	691	227
**V4**	0	0	0	0	0	0	0	0	0	0	0	0	0	0	0	0
**V5**	0	7663	34488	0	45915	45915	0	37285	0	840771	0	181990	13851	0	9346	11925
**V6**	0	8567	14436	66449	12535	72827	0	100799	42486	0	0	280578	5299	7838	24560	5744
**V7**	66788	8109	52254	0	10234	10234	1849	4789	10234	0	1176	109041	11183	41724	1992	2051
**V8**	40091	7714	15547	0	0	0	0	61434	0	317272	17720	0	0	0	16202	695
**V9**	0	4135	15453	0	10032	49794	10503	44525	23040	257840	0	269798	4846	7430	15070	0
**V10**	60402	0	30930	0	0	0	469	595	0	408482	0	13163	51594	39171	0	0
**V11**	0	0	37137	0	83888	31833	0	61514	25360	40617	0	161053	15580	24174	11201	0
**V12**	30720	6318	15820	83032	0	97560	16265	86752	0	18864	32838	215901	6398	10745	28581	2766
	**N18**	**N19**	**N20**	**N21**	**N22**	**N23**	**N24**	**N25**	**N26**	**N27**	**N28**	**N29**	**N30**	**N31**	**N32**	**N33**
**HHC**	28905	8295	17350	14677	54346	23139	42603	6703	20179	171391	5704	27438	28492	89055	27155	28609
**V1**	0	0	0	0	0	0	0	0	0	0	0	0	0	0	0	0
**V2**	28081	0	0	13321	35576	5668	14609	0	11269	82146	2533	14574	18251	55431	15736	14969
**V3**	8439	0	4199	12886	2853	0	2528	0	1085	12664	1312	1093	58053	58053	0	88303
**V4**	0	0	0	0	0	0	0	0	0	0	0	0	0	0	0	0
**V5**	0	0	36583	0	36430	12964	5900	0	41427	135192	4473	35527	18984	45403	39802	0
**V6**	31678	0	3586	7130	49461	14739	0	0	11770	169450	0	18770	38453	77926	19929	29118
**V7**	0	0	0	11461	9895	5689	898	2637	0	62795	2449	18195	0	0	14812	5925
**V8**	0	0	0	0	30610	7375	26760	0	3737	179358	5979	0	14354	101333	0	22213
**V9**	28198	0	0	5435	34609	0	0	0	39761	156179	4754	39241	26853	98361	41517	0
**V10**	0	0	0	17134	9810	0	0	0	0	22572	0	40467	0	0	31635	0
**V11**	30692	0	12395	69628	16398	0	7384	0	18833	86343	2694	16738	28172	73003	22219	19640
**V12**	0	0	2012	12692	65508	21346	12219	0	0	154538	0	8228	54424	90299	0	0

Table S1. Abundance of 68 putatively identified compounds in the LC-MS profile of the HHCF and its twelve varied formulae. (V1-V12 represented the twelve varied formulae. P1-P31 and N1-N37 represent compounds putatively identified in the HHCF LC-MS profile obtained under positive and negative ionization modes, respectively. The putatively assigned identity of P1-P31 and N1-N37 compounds can be found in Table 2a and 2b, respectively.)

	N34	N35	N36	N37
**HHC**	26925	41691	7390	69203
**V1**	0	0	0	0
**V2**	64009	0	24270	0
**V3**	0	0	0	0
**V4**	0	0	0	0
**V5**	14505	25189	16292	78503
**V6**	0	0	37845	40539
**V7**	7063	6786	3424	25123
**V8**	16633	45962	0	0
**V9**	0	30169	0	0
**V10**	12305	3275	0	10237
**V11**	0	0	10743	48133
**V12**	0	0	12606	19078

Table S2. Mean-centered and scaled abundance of 68 putatively identified compounds in the LC-MS profile of the HHCF and its twelve varied formulae. (V1-V12 represented the twelve varied formulae. P1-P31 and N1-N37 represent compounds putatively identified in the HHCF LC-MS profile obtained under positive and negative ionization modes, respectively. The putatively assigned identity of P1-P31 and N1-N37 compounds can be found in Table 2a and 2b, respectively.)

	P1	P2	P3	P4	P5	P6	P7	P8	P9	P10	P11	P12	P13	P14	P15	P16
**HHCF**	1.1391	0.5488	0.4492	0.6529	-0.0020	-0.2643	0.7209	0.9300	0.2626	0.0462	0.5825	0.8118	1.1094	1.6929	-0.1508	-0.0961
**V1**	-0.8380	-0.8958	-1.3051	-1.1869	-1.5607	2.4501	0.9470	-1.2269	-0.7067	0.4943	-0.4228	-0.7103	-0.5501	-0.5362	-1.8278	-1.7732
**V2**	-0.8380	-0.2346	0.9053	0.2586	1.1064	0.2417	1.1712	0.0305	1.1464	0.5317	0.8509	2.3729	-0.5501	-0.5573	0.5898	1.2738
**V3**	0.5963	-0.4702	0.2665	-0.7853	-0.5472	-0.4657	-1.4438	-1.0618	-0.5477	-0.7095	1.6555	-0.0597	-0.5501	-0.5573	-0.4762	0.1321
**V4**	0.9433	-0.8554	2.2512	0.3089	-0.1556	1.8381	1.6633	-1.3121	2.5192	3.0128	-0.8671	1.2389	-0.5501	-0.5573	0.9225	1.1803
**V5**	1.1936	0.1548	0.2742	0.3602	0.8686	0.0020	0.4595	0.5200	0.1675	-0.0185	0.2748	0.7810	0.6990	-0.5573	1.1374	1.0008
**V6**	-0.8380	-0.8958	-0.2843	-1.1869	0.2555	-0.4411	-0.3302	-0.6946	-0.6083	-0.4038	-1.1180	-0.1726	-0.5501	-0.5573	-0.1937	-0.1223
**V7**	1.7482	0.7818	0.9968	-1.1869	2.0950	-0.2297	0.3746	1.3761	0.6658	-0.1908	1.4256	-0.7103	-0.5501	-0.5573	1.3268	0.7480
**V8**	-0.8380	-0.8958	-0.5669	-1.1869	-0.1617	-0.5852	-0.0311	0.6579	-0.1737	-0.2874	0.5702	-0.7103	2.7157	2.0123	0.4732	0.2858
**V9**	0.2454	-0.5854	-0.6094	1.3490	-0.1153	-0.5810	-0.9351	-1.0038	-0.8200	-0.6324	-1.3148	-0.7103	-0.5501	-0.5573	-0.3633	-0.6323
**V10**	-0.8380	2.4627	-0.3212	0.2837	0.2812	-0.4683	-0.1839	1.3961	0.0153	-0.4110	-0.0450	-0.7103	-0.5501	1.5054	0.8007	0.5104
**V11**	-0.8380	1.0941	-0.8323	0.6913	-0.5033	-0.6907	-1.0044	0.7723	-0.7567	-0.6304	-0.2551	-0.7103	-0.5501	-0.5573	-0.5641	-0.8345
**V12**	-0.8380	-0.2090	-1.2240	1.6284	-1.5607	-0.8059	-1.4079	-0.3837	-1.1637	-0.8012	-1.3366	-0.7103	0.4269	-0.2161	-1.6745	-1.6730
	**P17**	**P18**	**P19**	**P20**	**P21**	**P22**	**P23**	**P24**	**P25**	**P26**	**P27**	**P28**	**P29**	**P30**	**P31**	**N1**
**HHCF**	-0.8094	0.6487	0.3119	0.7400	0.7764	0.7646	0.7196	1.3185	0.1455	0.0324	-0.1355	0.0497	-0.1309	-0.3603	0.0277	2.5564
**V1**	-1.1071	-0.8622	-1.1406	-1.0234	-1.2037	-1.0124	-0.9470	-0.6491	-0.6428	-1.0870	-0.6249	-0.4764	-0.7273	-0.5400	-1.4523	-0.4061
**V2**	-0.3035	-0.8702	1.4282	0.8052	1.9971	2.1966	2.2256	-0.6491	-0.0873	-0.0889	-0.3804	-0.4764	-0.7273	-0.4421	0.5082	1.9107
**V3**	0.5625	0.5246	0.0337	-0.2608	1.1787	-0.5126	-0.7287	-0.6491	-0.5486	-1.0352	-0.5148	-0.4478	-0.7066	-0.4661	-1.0242	-0.4061
**V4**	1.3706	1.3846	0.9943	2.0868	0.7067	-0.3813	-0.7459	-0.5676	-0.6428	2.4374	-0.5924	-0.4516	-0.5856	-0.5400	0.3807	-0.4061
**V5**	1.0438	0.8794	0.4592	0.2204	0.3074	0.8351	0.7728	-0.6491	-0.2975	-0.0600	-0.2727	-0.4764	-0.2574	-0.3072	0.1864	-0.4061
**V6**	1.9707	-0.8507	-1.1362	-0.9233	-0.0264	-0.7592	-0.7576	-0.6491	-0.6262	-0.8855	-0.5672	-0.4239	-0.6289	-0.5248	1.1195	-0.4061
**V7**	-0.5704	-0.8702	1.0123	1.0709	0.2740	1.2142	1.0963	1.6010	1.0559	0.6744	1.3680	3.1374	2.1344	2.5254	-1.4523	-0.4061
**V8**	-0.8328	1.2609	0.6947	0.4151	-0.3580	-0.0062	0.1980	-0.6491	0.0290	0.4953	-0.0245	0.5154	0.6240	0.0034	0.9311	-0.4061
**V9**	0.5416	-0.8583	-1.1491	-0.7586	-1.2037	-0.6607	-0.4443	0.7735	-0.5913	-0.8432	-0.5705	-0.4066	-0.6493	-0.5281	0.4929	-0.4061
**V10**	-0.8450	1.3444	0.7842	-0.1303	-0.3986	0.2699	0.4650	1.8972	2.9121	0.8974	2.8230	-0.4764	1.9762	1.8892	-1.4523	-0.4061
**V11**	-0.1158	-0.8629	-1.1434	-1.0569	-0.8462	-0.9355	-0.9126	-0.6491	-0.0629	0.3007	0.0089	0.3046	0.2249	-0.2376	0.4695	-0.4061
**V12**	-0.9052	-0.8679	-1.1491	-1.1850	-1.2037	-1.0124	-0.9413	-0.4790	-0.6428	-0.8379	-0.5171	-0.3717	-0.5463	-0.4718	1.2652	-0.4061

Table S2. Mean-centered and scaled abundance of 68 putatively identified compounds in the LC-MS profile of the HHCF and its twelve varied formulae. (V1-V12 represented the twelve varied formulae. P1-P31 and N1-N37 represent compounds putatively identified in the HHCF LC-MS profile obtained under positive and negative ionization modes, respectively. The putatively assigned identity of P1-P31 and N1-N37 compounds can be found in Table 2a and 2b, respectively.)

	N2	N3	N4	N5	N6	N7	N8	N9	N10	N11	N12	N13	N14	N15	N16	N17
**HHCF**	0.2875	0.2062	0.7697	0.4713	0.2780	0.3062	1.7976	0.4262	0.8506	2.0191	0.9365	0.6021	0.6693	1.1929	0.2441	1.9740
**V1**	-0.8840	-0.7560	-1.5033	-0.4840	-0.9444	-0.9262	-0.5524	-1.1114	-0.8339	-0.6709	-0.4969	-1.1547	-0.7880	-0.9278	-1.0545	-0.6572
**V2**	-0.0295	-0.7560	0.6127	-0.5375	0.3828	0.1488	-0.5524	-0.2491	0.8305	-0.6709	-0.4969	0.6261	-0.1699	-0.1353	0.0064	-0.2994
**V3**	1.9806	2.9743	0.7501	0.0572	1.8188	-0.9262	-0.5524	-0.7744	-0.4638	-0.6494	-0.4969	-1.0806	-0.5209	-0.4292	-0.9817	-0.6034
**V4**	-0.8840	-0.7560	-1.6009	-0.5375	-0.9444	-0.9262	-0.5524	-1.1114	-0.8339	-0.6709	-0.4969	-1.1547	-0.7880	-0.9278	-1.0545	-0.6572
**V5**	-0.8840	0.1698	0.5985	-0.5375	-0.0700	0.5240	-0.5524	-0.0134	-0.8339	2.0114	-0.4969	0.5299	0.2184	-0.9278	-0.0691	2.1726
**V6**	-0.8840	0.2791	-0.6803	1.8320	1.4428	1.3740	-0.5524	1.8569	2.1583	-0.6709	-0.4969	1.4425	-0.4030	-0.4106	1.5350	0.7059
**V7**	1.3428	0.2237	1.7315	-0.5375	-0.7495	-0.6030	-0.2453	-0.9704	-0.1131	-0.6709	-0.3826	-0.1454	0.0245	1.8254	-0.8445	-0.1705
**V8**	0.4527	0.1760	-0.6094	-0.5375	-0.9444	-0.9262	-0.5524	0.6977	-0.8339	0.3413	1.2247	-1.1547	-0.7880	-0.9278	0.6537	-0.4923
**V9**	-0.8840	-0.2564	-0.6154	-0.5375	0.9662	0.6465	1.1923	0.1998	0.7887	0.1517	-0.4969	1.3427	-0.4359	-0.4375	0.5344	-0.6572
**V10**	1.1299	-0.7560	0.3716	-0.5375	-0.9444	-0.9262	-0.4745	-1.0939	-0.8339	0.6323	-0.4969	-1.0329	2.9607	1.6570	-1.0545	-0.6572
**V11**	-0.8840	-0.7560	0.7674	-0.5375	0.6531	0.0792	-0.5524	0.7001	0.9521	-0.5413	-0.4969	0.3361	0.3440	0.6674	0.1265	-0.6572
**V12**	0.1402	0.0073	-0.5920	2.4234	-0.9444	2.1552	2.1495	1.4433	-0.8339	-0.6107	2.6934	0.8438	-0.3231	-0.2188	1.9589	-0.0008

	N18	N19	N20	N21	N22	N23	N24	N25	N26	N27	N28	N29	N30	N31	N32	N33
**HHCF**	1.1565	3.3282	1.0712	-0.3315	1.2733	1.9078	2.6208	3.0845	0.5911	1.0944	1.4796	0.6990	0.3264	0.8963	0.6914	0.5106
**V1**	-0.8209	-0.2774	-0.5457	-0.6053	-1.2186	-0.8265	-0.6711	-0.3703	-0.7659	-1.3551	-0.9996	-1.1285	-1.1067	-1.3168	-1.0493	-0.6534
**V2**	1.1002	-0.2774	-0.5457	-0.3568	0.4126	-0.1567	0.4578	-0.3703	-0.0081	-0.1811	0.1013	-0.1578	-0.1887	0.0607	-0.0406	-0.0444
**V3**	-0.2436	-0.2774	-0.1544	-0.3649	-1.0878	-0.8265	-0.4757	-0.3703	-0.6930	-1.1741	-0.4293	-1.0557	1.8133	0.1258	-1.0493	2.9393
**V4**	-0.8209	-0.2774	-0.5457	-0.6053	-1.2186	-0.8265	-0.6711	-0.3703	-0.7659	-1.3551	-0.9996	-1.1285	-1.1067	-1.3168	-1.0493	-0.6534
**V5**	-0.8209	-0.2774	2.8635	-0.6053	0.4518	0.7055	-0.2152	-0.3703	2.0200	0.5770	0.9445	1.2377	-0.1518	-0.1885	1.5021	-0.6534
**V6**	1.3462	-0.2774	-0.2115	-0.4723	1.0493	0.9152	-0.6711	-0.3703	0.0256	1.0666	-0.9996	0.1216	0.8274	0.6197	0.2282	0.5313
**V7**	-0.8209	-0.2774	-0.5457	1.5333	-0.7649	-0.1542	-0.6017	0.9888	-0.7659	-0.4577	0.0648	0.0833	-1.1067	-1.3168	-0.0998	-0.4123
**V8**	-0.8209	-0.2774	-0.5457	-0.6053	0.1849	0.0450	1.3967	-0.3703	-0.5146	1.2082	1.5991	-1.1285	-0.3847	1.2014	-1.0493	0.2504
**V9**	1.1082	-0.2774	-0.5457	-0.5039	0.3683	-0.8265	-0.6711	-0.3703	1.9080	0.8770	1.0667	1.4851	0.2440	1.1275	1.6120	-0.6534
**V10**	-0.8209	-0.2774	-0.5457	2.5920	-0.7688	-0.8265	-0.6711	-0.3703	-0.7659	-1.0325	-0.9996	1.5667	-1.1067	-1.3168	0.9785	-0.6534
**V11**	1.2788	-0.2774	0.6094	0.6939	-0.4667	-0.8265	-0.1005	-0.3703	0.5006	-0.1211	0.1713	-0.0137	0.3103	0.4973	0.3750	0.1457
**V12**	-0.8209	-0.2774	-0.3582	-0.3685	1.7851	1.6960	0.2731	-0.3703	-0.7659	0.8535	-0.9996	-0.5805	1.6307	0.9272	-1.0493	-0.6534

Table S2. Mean-centered and scaled abundance of 68 putatively identified compounds in the LC-MS profile of the HHCF and its twelve varied formulae. (V1-V12 represented the twelve varied formulae. P1-P31 and N1-N37 represent compounds putatively identified in the HHCF LC-MS profile obtained under positive and negative ionization modes, respectively. The putatively assigned identity of P1-P31 and N1-N37 compounds can be found in Table 2a and 2b, respectively.)

	**N34**	**N35**	**N36**	**N37**
**HHCF**	0.8820	1.7154	-0.1082	1.1202
**V1**	-0.5980	-0.6752	-0.7379	-0.8303
**V2**	2.9204	-0.6752	1.3302	-0.8303
**V3**	-0.5980	-0.6752	-0.7379	-0.8303
**V4**	-0.5980	-0.6752	-0.7379	-0.8303
**V5**	0.1993	0.7692	0.6504	1.3823
**V6**	-0.5980	-0.6752	2.4870	0.3123
**V7**	-0.2098	-0.2861	-0.4461	-0.1222
**V8**	0.3162	1.9603	-0.7379	-0.8303
**V9**	-0.5980	1.0547	-0.7379	-0.8303
**V10**	0.0783	-0.4874	-0.7379	2.0550
**V11**	-0.5980	-0.6752	0.1776	0.5264
**V12**	-0.5980	-0.6752	0.3363	-0.2925

Table S3. The levels of CCL17 produced by TNF-α plus IFN-ϒ-stimulated HaCaT after treatment with the HHCF and its twelve varied formulae decoctions at a concentration of 60 μg/ml.

	**Raw data [CCL17 levels (% of control)]**	**Reciprocal of raw data**	**Mean-centered and scaled data**
**HHCF**	58.602	1.706	-0.796
**V1**	71.857	1.392	-0.982
**V2**	50.617	1.976	-0.637
**V3**	30.587	3.269	0.128
**V4**	61.641	1.622	-0.846
**V5**	46.571	2.147	-0.536
**V6**	20.562	4.863	1.070
**V7**	63.006	1.587	-0.867
**V8**	24.785	4.035	0.580
**V9**	19.365	5.164	1.248
**V10**	56.446	1.772	-0.758
**V11**	32.249	3.101	0.028
**V12**	14.165	7.060	2.369

Table S4. Percentage of variance explained by the PLS-R-model.

	**X Variables**		**Y Variables**	
**LV**	**This LV**	**Total**	**This LV**	**Total**
1	23.97	23.97	79.69	79.69
2	9.69	33.66	14.54	94.23
3	9.76	43.42	0.58	98.89
4	13.34	56.76	0.41	99.48
5	8.46	65.22	0.08	99.89
6	6.81	72.04	0.03	99.96
7	4.22	76.26	0.00	99.99
8	5.76	82.01	0.00	100.00
**9**	**6.18**	**88.19**	**0.00**	**100.00**
10	3.98	92.17	0.00	100.00
11	3.89	96.06	0.00	100.00
12	3.94	100.00	0.00	100.00

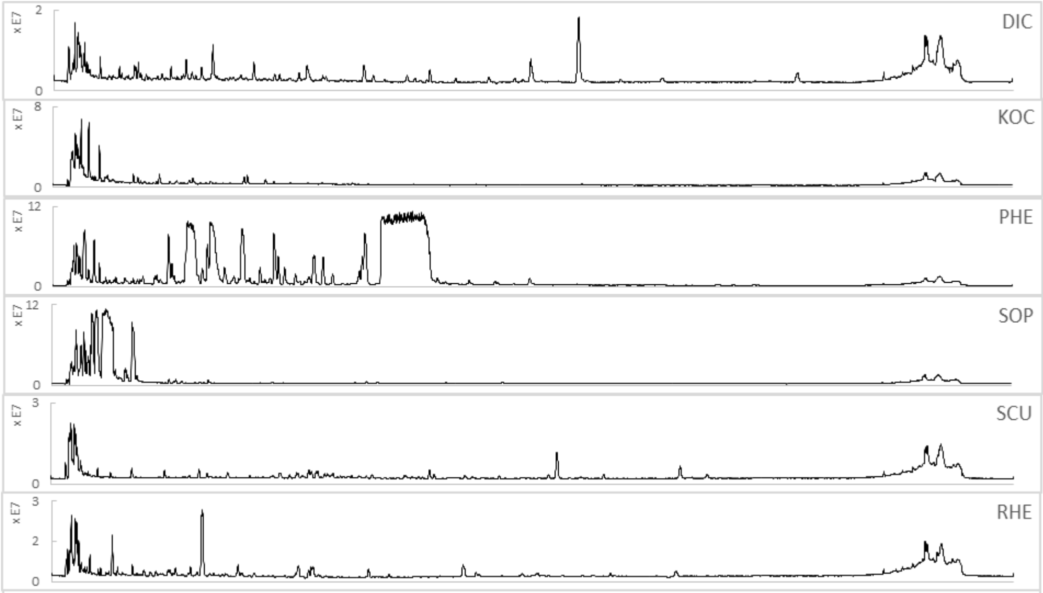

Figure S1. TICs of DIC, KOC, PHE, SOP, SCU and RHE in positive ionization mode.

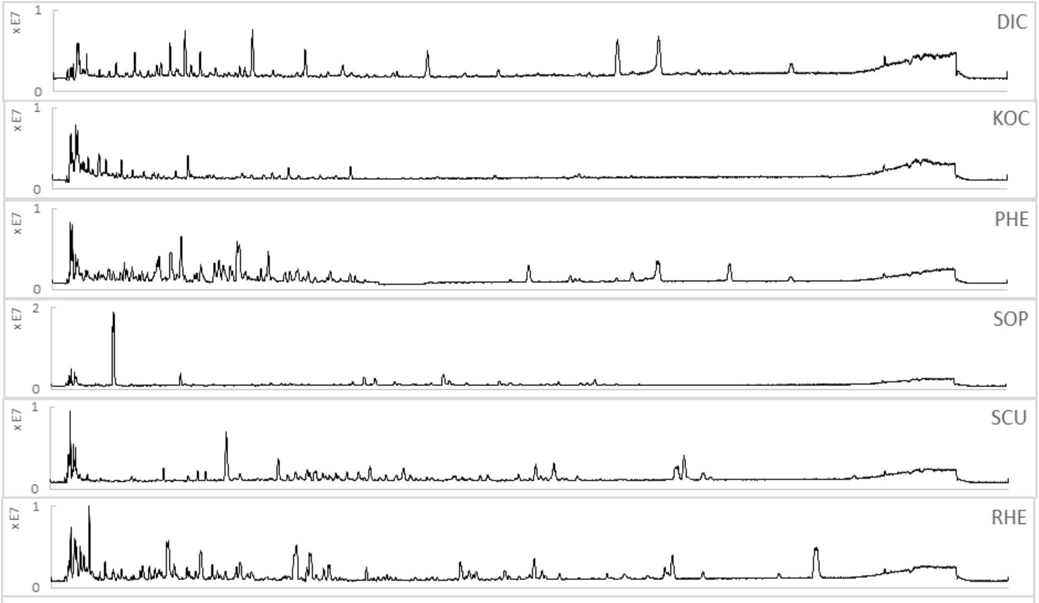

Figure S2. TICs of DIC, KOC, PHE, SOP, SCU and RHE in negative ionization mode.

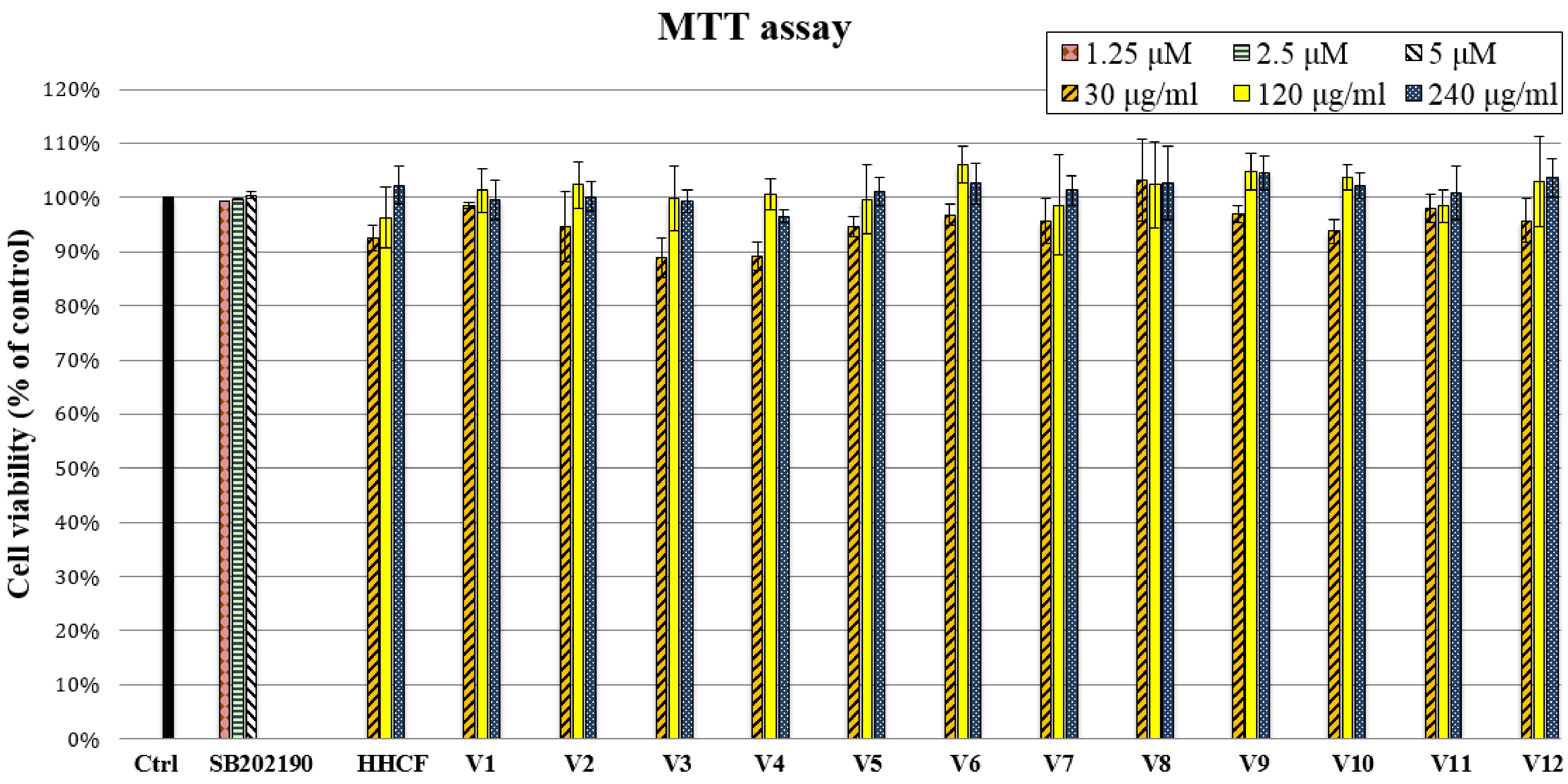

Figure S3. Effect of the HHCF and its twelve varied formulae (V1-V12) (30, 120 and 240 µg/ml) and SB202190 monohydrochloride hydrate (1.25, 2.5 and 5 µM) on the viability of HaCaT. Data are represented as mean ± standard error of three independent experiments (n=3). Statistical significance was determined using one-way analysis of variance with Dunnett's multiple comparisons test. * $p<0.05$ versus control (Ctrl).

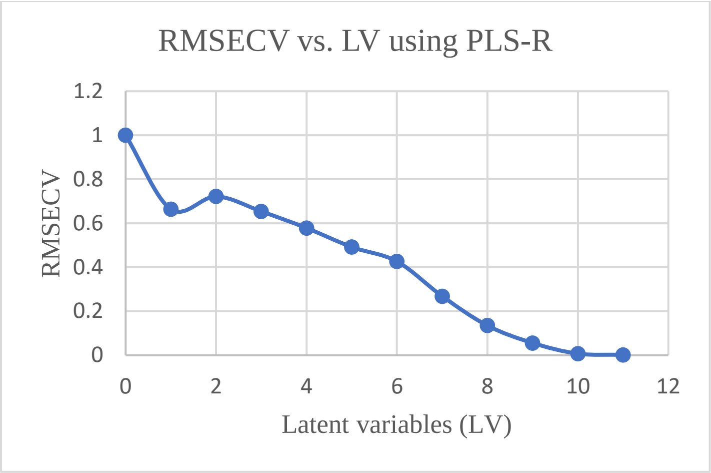

Figure S4. Root-mean-square error of cross-validation (RMSECV) versus latent factors included in the PLS-R analysis.
